# XAF1 promotes neuroblastoma tumor suppression and is required for KIF1Bβ-mediated apoptosis

**DOI:** 10.18632/oncotarget.8748

**Published:** 2016-04-15

**Authors:** Zhang'e Choo, Rachel Yu Lin Koh, Karin Wallis, Timothy Jia Wei Koh, Chik Hong Kuick, Veronica Sobrado, Rajappa S. Kenchappa, Amos Hong Pheng Loh, Shui Yen Soh, Susanne Schlisio, Kenneth Tou En Chang, Zhi Xiong Chen

**Affiliations:** ^1^ Department of Physiology, Yong Loo Lin School of Medicine, National University of Singapore, S117597, Singapore, Singapore; ^2^ Ludwig Cancer Research (Stockholm), Karolinska Institutet, SE-17177, Stockholm, Sweden; ^3^ School of Life Sciences and Technology, Ngee Ann Polytechnic, S599489, Singapore, Singapore; ^4^ Department of Pathology and Laboratory Medicine, KK Women's and Children's Hospital, S299899, Singapore; ^5^ Neuro-Oncology Program, Moffitt Cancer Center, Tampa, FL 33612, USA; ^6^ Department of Paediatric Surgery, KK Women's and Children's Hospital, S299899, Singapore, Singapore; ^7^ Department of Paediatric Hematology/Oncology, KK Women's and Children's Hospital, S299899, Singapore, Singapore; ^8^ Department of Microbiology and Tumor and Cell Biology, Karolinska Institutet, 17177 Stockholm, Sweden

**Keywords:** XAF1, neuroblastoma, KIF1Bβ, apoptosis

## Abstract

Neuroblastoma is an aggressive, relapse-prone childhood tumor of the sympathetic nervous system. Current treatment modalities do not fully exploit the genetic basis between the different molecular subtypes and little is known about the targets discovered in recent mutational and genetic studies. Neuroblastomas with poor prognosis are often characterized by 1p36 deletion, containing the kinesin gene *KIF1B.* Its beta isoform, KIF1Bβ, is required for NGF withdrawal-dependent apoptosis, mediated by the induction of XIAP-associated Factor 1 (XAF1). Here, we showed that XAF1 low expression correlates with poor survival and disease status. KIF1Bβ deletion results in loss of XAF1 expression, suggesting that XAF1 is indeed a downstream target of KIF1Bβ. XAF1 silencing protects from NGF withdrawal and from KIF1Bβ-mediated apoptosis. Overexpression of XAF1 impairs tumor progression whereas knockdown of XAF1 promotes tumor growth, suggesting that XAF1 may be a candidate tumor suppressor in neuroblastoma and its associated pathway may be important for developing future interventions.

## INTRODUCTION

Neuroblastoma is a neural crest-derived embryonal tumor of the sympathetic nervous system. It is the most common extracranial childhood solid tumor that accounts for 15% of pediatric cancer deaths [1, 2]. One of the mechanisms that give rise to neuroblastoma is the failure of neural progenitors to undergo apoptosis via an EglN3-dependent pathway when nerve growth factor (NGF) becomes limiting during development [3]. This form of developmental apoptosis is important for maintaining an appropriate number of terminally differentiated cells, the failure of which may lead to excessive progenitor cells and increase the likelihood of subsequent tumor development [4, 5]. Indeed, mutations of several genes implicated along the EglN3-dependent pathway have been found to associate with neuroblastoma and malignancies of neural crest origin such as paraganglioma and pheochromocytoma [3, 6, 7].

KIF1Bβ is a kinesin that was identified as the key player mediating the pro-apoptotic effects downstream of EglN3 in this developmental culling pathway. *KIF1B* gene resides on chromosome 1p36.2, a region frequently deleted in neuroblastomas [8]. Recently, it was found that KIF1Bβ interacts with RNA helicase A (DHX9), which increases the nuclear translocation and accumulation of DHX9, and correlates with increased mRNA of pro-apoptotic XIAP-associated factor 1 (XAF1) [9].

XAF1 is a potent antagonist of anti-apoptotic XIAP and Survivin whereby Survivin is associated with high-risk neuroblastoma [10, 11]. Interestingly, previous work has shown that removal of XIAP is required for sympathetic neurons to gain ‘apoptotic competence’ prior to growth factor withdrawal-induced apoptosis [12]. Studies also showed that loss or reduction of XAF1 expression without any corresponding inactivating mutation or deletion, is frequently associated with malignant tumor progression in a variety of cancers including melanoma, gastric, colon and pancreatic cancers [13–18]. Restoration of XAF1 expression was found to induce apoptosis and inhibit tumor growth in these cancers, implicating XAF1 as a candidate tumor suppressor [15–18]. This reinforced the notion that upstream regulation of XAF1 by KIF1Bβ could be a relevant tumor suppression pathway in neuroblastoma.

Despite studies implicating XAF1 as a potential tumor suppressor in several cancers, the role of XAF1 in neuroblastoma tumor suppression remains unknown. In this study, we investigated the expression of XAF1 as well as its role in tumor suppression and apoptosis induction in neuroblastoma.

## RESULTS

### XAF1 is poorly expressed in neuroblastoma tumors and KIF1Bβ-deficient mouse sympathetic neuroblasts

**Figure 1:**
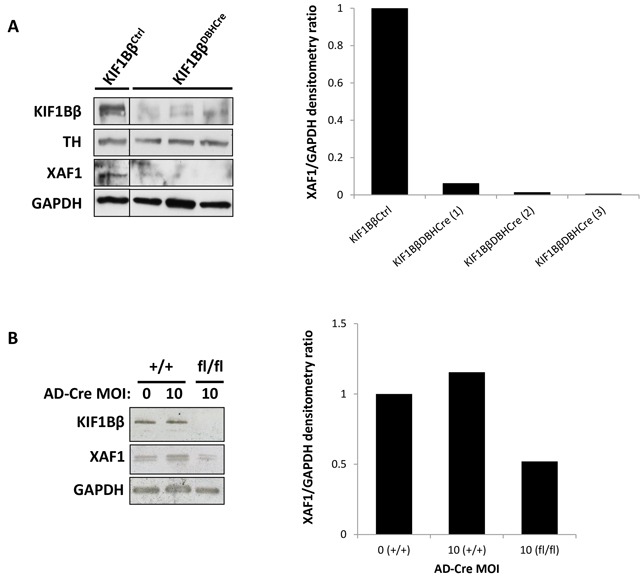
XAF1 is poorly expressed in KIF1Bβ Knock-out mouse sympathetic neuroblasts **A.** Immunoblot analysis of isolated primary sympathetic neuroblasts from superior cervical ganglia of wild type and conditional KIF1Bβ Knock-out (KO) mouse. Right – corresponding densitometry for XAF1 expression. **B.** Immunoblot analysis of isolated primary sympathetic neuroblasts deleted for KIF1Bβ *ex* vivo by treatment with Cre Recombinase adenovirus. Right – corresponding densitometry for XAF1 expression.

To determine the correlation between the expression of KIF1Bβ and XAF1, we used a conditional KIF1Bβ Knock-Out (KO) mouse under DBH-Cre control to specifically delete *KIF1Bβ* in primary sympathetic neuroblasts from the superior cervical ganglia (SCG) of post-gestational day 0-2 (P0 – P2) mouse pups and then analyzed for XAF1 protein expression. Protein analysis showed a significant reduction in XAF1 expression corresponding to KIF1Bβ deletion (Figure 1A). Similarly, when *KIF1Bβ* flanked by LoxP sequences was deleted *ex vivo* by treating isolated mouse primary sympathetic neuroblasts in culture with adenovirus expressing Cre recombinase, XAF1 protein expression was also correspondingly reduced, suggesting that XAF1 is a downstream target of KIF1Bβ (Figure 1B). Moreover, bioinformatics analysis from NextBio database revealed that KIF1Bβ was poorly expressed in adult SCG as compared to other tissues of the human nervous system (Supplementary Figure S1A). Intriguingly but not unexpectedly, XAF1 was also poorly expressed in adult SCG (Supplementary Figure S1B).

**Figure 2:**
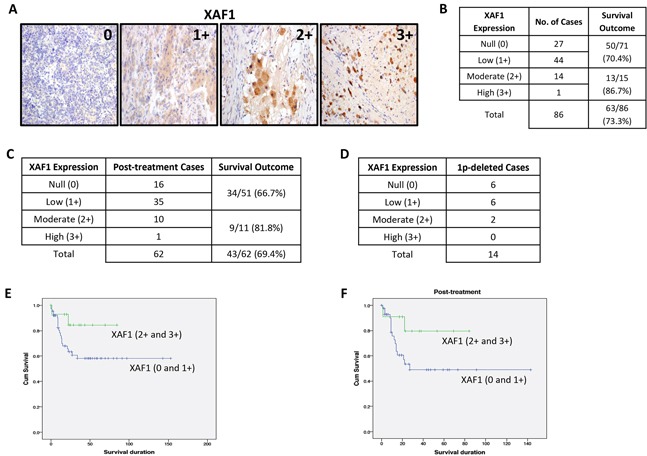
XAF1 is poorly expressed in neuroblastomas **A.** XAF1 immunohistochemistry staining in a tissue microarray (TMA) (n=86). Images are acquired using a 20X objective. Staining intensity grade is indicated in the upper right corner of representative images shown; 0=null, 1+=low, 2+=moderate, 3+=high. Images of 2+ and 3+ XAF1 staining depict post-treatment neuroblastic tumors. **B.** Association between XAF1 expression and survival outcome for neuroblastoma patients. **C.** Association between XAF1 expression and survival outcome for post-treatment neuroblastoma patients. **D.** Distribution of XAF1 expression in 1p-deleted neuroblastoma patients. **E. and F.** Kaplan-Meier survival curves for patients with complete absence and low expression of XAF1 (0 and 1+) compared to moderate and high expression of XAF1 (2+ and 3+) – (E) Total number of cases and (F) post-treatment cases.

Next, we examined the prognostic value of XAF1 expression using a tissue microarray (TMA) of human neuroblastomas, and performed survival analyses stratified by treatment status (Figure 2A–2F, Supplementary S2A–S2C and Supplementary Table S1). According to TMA analysis, the median 5-year overall survival of all 86 patients is 73.3% (63 out of 86 cases) and majority of neuroblastomas (71 out of 86 cases) showed null (0) or low (1+) expression of XAF1 (Figure 2B). Notably, neuroblastoma patients with moderate (2+) or high (3+) expression of XAF1 (15 out of 86 cases) showed a better survival outcome (86.7%, 13 out of 15 cases) as compared to those with complete loss or low XAF1 expression (70.4%, 50 out of 71 cases), suggesting a possible correlation between XAF1 expression and survival outcome (Figure 2B). Furthermore, 38 out of 86 patients are alive with known disease status and classified as either alive with disease (AWD) or showed no evidence of disease (NED) (Supplementary Figure S2A). Among these 38 patients, 6 out of 7 AWD cases showed complete loss or low expression of XAF1 (Supplementary Figure S2A), indicating that XAF1 expression may also be associated with the disease status and quality of survival. Similarly, analysis of post-treatment cases (62 out of 86 cases) revealed that post-treatment cases with moderate or high expression of XAF1 showed a better survival outcome (81.8%, 9 out of 11 cases) compared to those with null or low XAF1 expression (66.7%, 34 out of 51 cases) (Figure 2C). Among these 62 post-treatment cases, 22 patients are alive with known disease status (Supplementary Figure S2B). Out of these 22 patients, there are 4 AWD cases and all of them showed complete loss or low expression of XAF1 (Supplementary Figure S2B). Furthermore, among 14 cases with chromosome 1p deletion, 12 of them showed complete loss or low XAF1 expression (Figure 2D). Taken together, this suggests that XAF1 expression may be indicative for survival outcome and disease status, and treatments that restore XAF1 expression or its associated pathway may be a promising approach to overcome neuroblastoma. Indeed, low or complete absence of XAF1 expression (0 and 1+) is correlated with poor prognosis and reduced survival of neuroblastoma patients compared to moderate or high XAF1 expression (2+ and 3+) as indicated by the Kaplan-Meier curves, providing further evidence to suggest that XAF1 may be a potential tumor suppressor (Figure 2E, 2F and Supplementary S2C).

### XAF1 overexpression induces apoptosis in neuroblastoma cells

**Figure 3:**
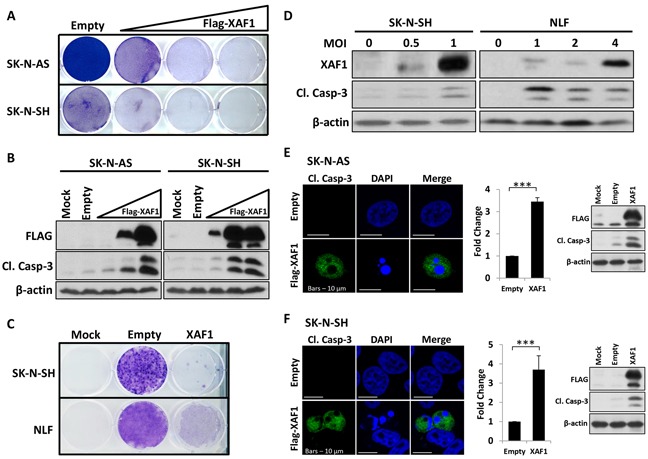
Overexpression of XAF1 induces apoptosis in neuroblastoma cells **A.** Crystal violet staining to determine cell viability in SK-N-AS (top) and SK-N-SH (bottom) after transfection with an increasing dose of 1 μg, 2 μg and 4 μg Flag-XAF1 plasmid. Transfected cells were selected with 2 mg/ml G418 (SK-N-AS) and 500 μg/ml G418 (SK-N-SH) for several weeks. Empty vector (empty) served as negative control. **B.** Corresponding immunoblot analysis of SK-N-AS and SK-N-SH cells. **C.** Crystal violet staining of SK-N-SH (top) and NLF (bottom) cells that were transduced with lentivirus encoding XAF1 and selected with 1μg/ml puromycin for several weeks. Control virus (empty) served as negative control. **D.** Immunoblot analysis of SK-N-SH and NLF cell lines transduced with increasing MOI of lentivirus encoding XAF1. **E. and F.** Anti-cleaved caspase-3 (green) immunofluorescence staining of SK-N-AS (E) and SK-N-SH (F) 24 hours post-transfection with plasmid encoding Flag-XAF1 or empty vector. Cells were counterstained with DAPI to visualize nuclei (blue). Scale bar – 10 μm. Right – graphical representation showing fold change in number of cleaved caspase-3-positive cells transfected with Flag-XAF1 relative to empty vector-transfected cells (mean ± SD; n=3; ***, P<0.001) and corresponding immunoblot analysis.

To determine the effect of XAF1 on neuroblastoma *in vitro*, we transfected SK-N-AS and SK-N-SH neuroblastoma cells with increasing expression of XAF1 and stained cells with crystal violet to determine cell viability (Figure 3A). Firstly, a panel of neuroblastoma and neural crest-derived melanoma cell lines were screened for XAF1 basal expression including SK-N-AS and SK-N-SH neuroblastoma cells (Supplementary Figure S3A). Ectopic expression of XAF1 was observed to decrease the colony formation of cells over time in a dose-dependent manner (Figure 3A). Overexpression of XAF1 was also found to induce apoptosis, as determined by caspase-3 cleavage (Figure 3B). Alternatively, we used a lentiviral approach to overexpress XAF1 in SK-N-SH and NLF neuroblastoma cells and similar results were obtained (Figure 3C and 3D). In addition, we quantified apoptosis by scoring the apoptotic nuclei of neuroblastoma cells expressing GFP-histone fusion protein (Figure S3B and S3C). In SK-N-AS and SK-N-SH cells co-transfected with GFP-histone and empty vector, approximately 5-10% of basal cell death was observed. However, cell death increased to 29-55% in SK-N-AS and 22-29% in SK-N-SH when GFP-histone was co-expressed with XAF1. In both cell lines, apoptosis was shown to peak at 24 hours post-transfection with XAF1 (Supplementary Figure S3B). Next, we also assessed apoptosis that resulted from XAF1 overexpression via immunofluorescence staining of cleaved caspase-3. In SK-N-AS and SK-N-SH neuroblastoma cells transfected with empty vector control, the nuclei were healthy and uniform with no cleaved caspase-3 detected whereas cells transfected with XAF1 displayed signs of apoptosis - nuclear condensation and fragmentation accompanied by the presence of cleaved caspase-3 (Figure 3E and 3F). Quantification of cleaved caspase-3 showed that cells with XAF1 overexpression exhibit significant increase in apoptosis (Figure 3E and 3F). Moreover, XAF1 overexpression was also able to induce apoptosis to a similar or greater extent than KIF1Bβ alone in NB1 neuroblastoma cells and neural crest-derived SK-MEL-28 melanoma cells (Supplementary Figure S3C). Taken together, these results demonstrate that XAF1 overexpression is sufficient to induce apoptosis in neuroblastoma cells *in vitro*.

### XAF1 is necessary for KIF1Bβ-mediated and NGF withdrawal-induced apoptosis

**Figure 4:**
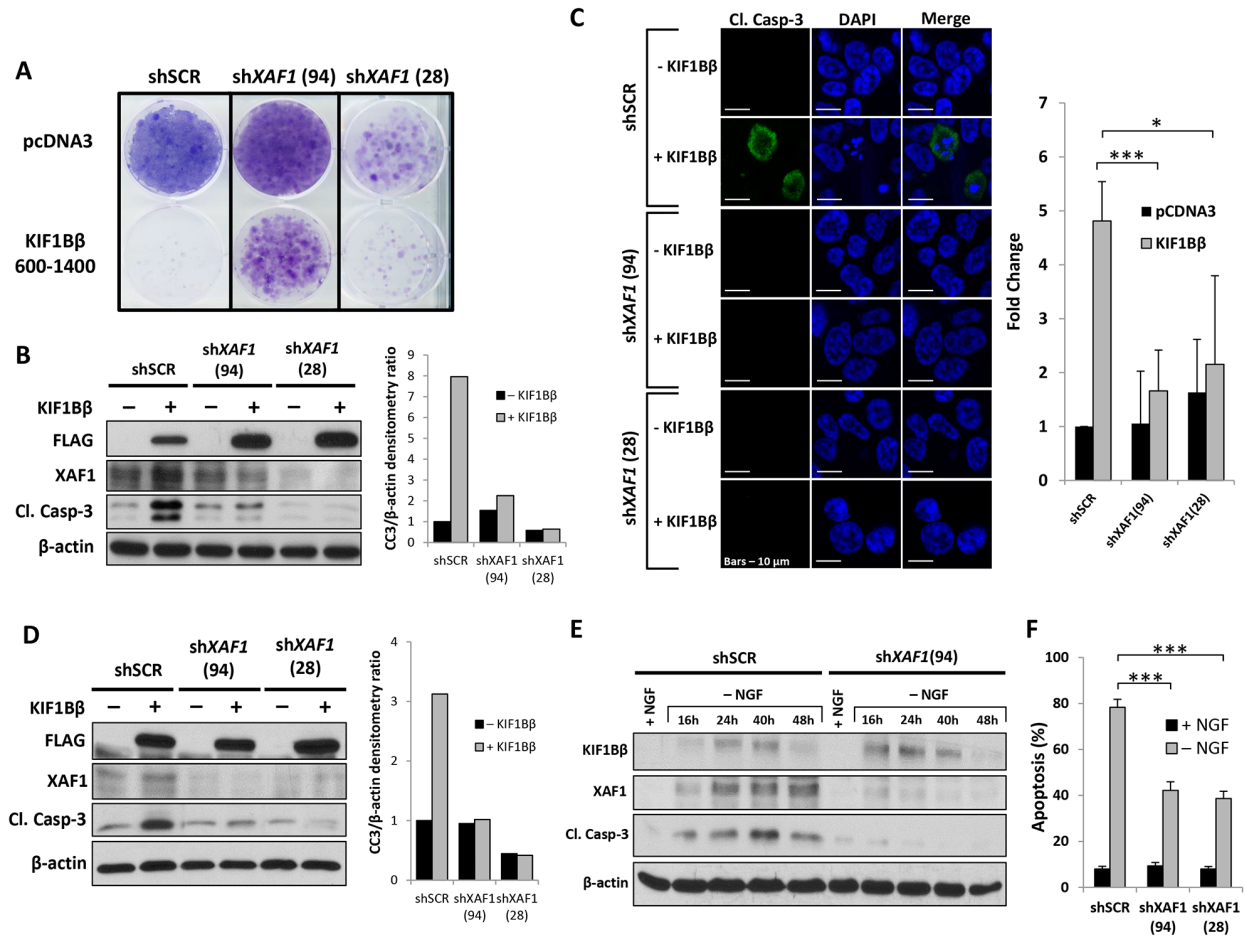
XAF1 is required for KIF1Bβ-mediated and NGF withdrawal-dependent apoptosis **A.** Crystal violet staining to determine cell viability in CHP212 cells after transduction with lentivirus encoding shRNAs targeting XAF1 (shXAF1 94 and 28) or non-targeting control virus (shSCR) and transfected with either Flag-KIF1Bβ(600-1400) or control empty pcDNA3 plasmids. Transfected cells were selected with 500 μg/ml G418 for several weeks. **B.** Corresponding immunoblot analysis of CHP212 cells. Right – densitometry for cleaved caspase-3 (CC3) expression. **C.** Anti-cleaved caspase-3 (green) immunofluorescence staining of CHP212 cells stably expressing shRNAs targeting XAF1 (shXAF1 94 and 28) or non-targeting control (shSCR) followed by transfection with Flag-KIF1Bβ(600-1400) or empty pcDNA3 plasmids as indicated. Cells were counterstained with DAPI to visualize nuclei (blue). Scale bar – 10 μm. Right -graphical representation showing fold change in number of cleaved caspase-3-positive CHP212 cells transduced with shRNAs targeting XAF1 (shXAF1 94 and 28) and transfected with either Flag-KIF1Bβ(600-1400) or empty pcDNA3 plasmids, relative to cells transduced with non-targeting control (shSCR) (mean ± SD; n=4; *, P<0.05; ***, P<0.001). **D.** Immunoblot analysis of NB1 cells stably transduced with shRNAs lentivirus targeting XAF1 (shXAF1 94 and 28) or control virus (shSCR), followed by transfection with either Flag-KIF1Bβ(600-1400) or control empty pcDNA3 plasmids. Right – corresponding densitometry for CC3 expression. **E.** Immunoblot analysis of differentiated PC12 cells before (+) and after (−) NGF withdrawal as indicated. Differentiated cells were transduced with lentivirus encoding shRNA targeting XAF1 (shXAF1 94) or non-targeting control virus (shSCR) prior to NGF withdrawal. **F.** Percentage of apoptosis in isolated primary rat sympathetic neuroblasts before (+) and after (−) NGF withdrawal. Neuroblasts were transduced with shRNAs lentivirus targeting XAF1 (shXAF1 94 and 28) or control virus (shSCR) prior to NGF withdrawal. Apoptosis was scored by apoptotic nuclei quantification in Tuj1-positive neuroblasts (mean ± SD; n=3; ***, P<0.001).

Next, we asked whether XAF1 is required for KIF1Bβ-mediated apoptosis in neuroblastoma. To do so, we used a high XAF1-expressing CHP212 neuroblastoma cells (Supplementary Figure S3A). Knockdown of XAF1 in CHP212 cells using lentiviral shRNAs targeting XAF1 transcript (shXAF1 94 and 28) conferred partial protection from apoptosis induced by ectopic expression of apoptotic KIF1Bβ(600-1400) domain compared to non-targeting shRNA (shSCR) control as determined by crystal violet staining for cell viability and colony formation [9] (Figure 4A). Similarly, knockdown of XAF1 with two independent hairpins (94 and 28) resulted in decreased levels of cleaved caspase-3 and number of cleaved caspase-3-positive CHP212 cells in the presence of KIF1Bβ compared to shSCR control (Figure 4B and 4C). Similarly, using KIF1Bβ homozygous-deleted NB1 neuroblastoma cell line, a decrease in cleaved caspase-3 levels upon ectopic expression of KIF1Bβ was also observed in the presence of XAF1 knockdown (Figure 4D). Taken together, these results suggest that XAF1 silencing protects against KIF1Bβ-mediated apoptosis and that XAF1 is necessary for KIF1Bβ apoptotic pathway in neuroblastoma cells. In order to determine whether XAF1 is necessary for NGF withdrawal-induced apoptosis, we made use of rat pheochromocytoma PC12 cells and primary sympathetic neuroblasts isolated from the SCGs of post-gestation rat pups (P2) as a model for NGF withdrawal-dependent apoptosis. Consistent with previous reports, NGF deprivation in differentiated PC12 cells resulted in the synchronized induction of KIF1Bβ and XAF1, and increased apoptosis as determined by cleaved caspase-3 [8, 9] (Figure 4E). Importantly, we observed that knockdown of XAF1 protected PC12 cells from apoptosis despite the up-regulation of KIF1Bβ expression upon NGF withdrawal (Figure 4E). Similarly, isolated primary sympathetic neuroblasts transduced with shRNAs targeting XAF1 demonstrated a significant reduction in apoptosis upon NGF withdrawal compared with shSCR control (Figure 4F). This suggests that XAF1 is a downstream effector specific to KIF1Bβ and is necessary for NGF withdrawal-dependent apoptosis.

### XAF1 expression determines neuroblastoma tumor growth *in vivo*

**Figure 5:**
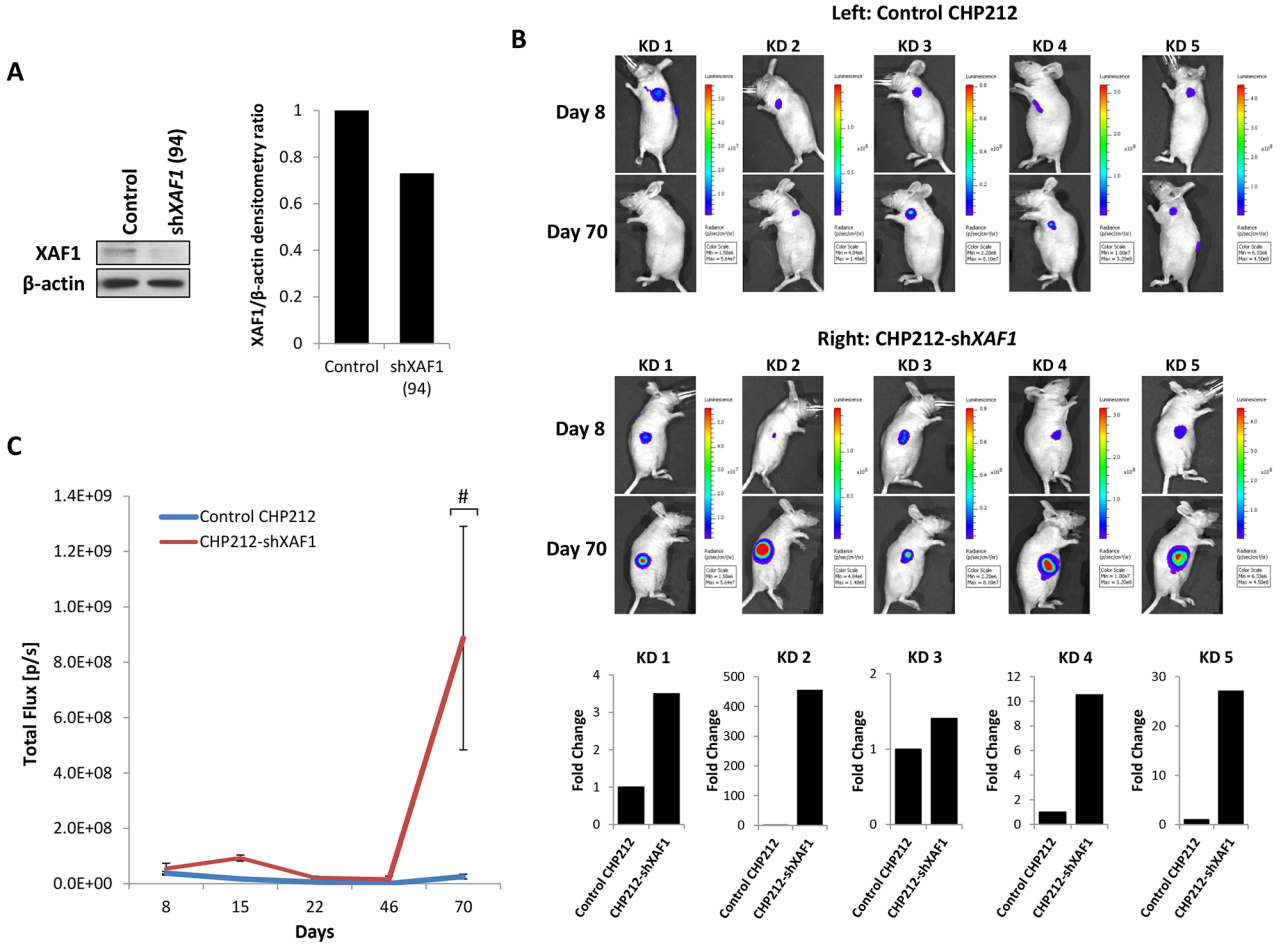
XAF1 knockdown promotes tumor progression *in vivo* **A.** Anti-XAF1 immunoblot analysis of CHP212 cells with (shXAF1 94) or without (Control) stable XAF1 knockdown and corresponding densitometry. **B.** Bioluminescence imaging of five representative nude mice (KD 1-5) on Day 8 and 70. Each mouse was injected with control CHP212 and CHP212-shXAF1 neuroblastoma cells on the left and right flanks respectively. Bottom – corresponding graphical representation (KD 1-5) showing fold change in tumor growth generated by CHP212-shXAF1 cells relative to control CHP212 cells. **C.** Tumor growth analysis by quantifying bioluminescent signal in nude mice over a period of 70 days (mean ± SEM; n=5; #, P=0.05).

To test the potential significance of XAF1 expression on tumor growth *in vivo*, we investigated the effects of XAF1 on tumor growth using mouse xenograft models. Firstly, CHP212 cells with and without stably-expressed XAF1 knockdown were generated as verified via Western Blot (Figure 5A) and subsequently engrafted subcutaneously into the right and left flanks of nude mice on day 0 respectively. Tumor growth was monitored over a period of 70 days and was determined by quantifying bioluminescence signal expressed as total flux (photons per sec) (Figure 5B and 5C). A comparable and modest tumor growth was initially observed in the XAF1 knockdown and control groups over a period of 46 days (Figure 5C). Subsequently, XAF1 knockdown group showed a distinct increase in tumor growth rate compared to the control group, suggesting that XAF1 knockdown promotes neuroblastoma tumor growth *in vivo* (Figure 5B and 5C).

**Figure 6:**
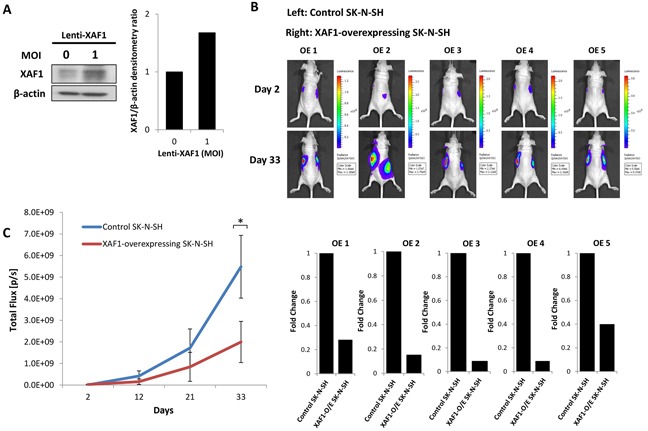
XAF1 overexpression delays tumor progression *in vivo* **A.** Anti-XAF1 immunoblot analysis of SK-N-SH cells transduced with or without XAF1-overexpressing lentivirus and corresponding densitometry. **B.** Bioluminescence imaging of five representative nude mice (OE 1-5) on day 2 and 33. Each mouse was engrafted with control SK-N-SH and XAF1-overexpressing SK-N-SH neuroblastoma cells on the left and right flanks respectively. Bottom – corresponding graphical representation (OE 1-5) showing fold change in tumor growth generated by XAF1-overexpressing SK-N-SH cells relative to control SK-N-SH cells. **C.** Tumor growth analysis by quantifying bioluminescent signal in nude mice over a period of 33 days (mean ± SEM; n=5; *, P<0.05).

To assess the effect of XAF1 overexpression on neuroblastoma tumor growth *in vivo*, we generated SK-N-SH neuroblastoma cells transduced with lentivirus to stably overexpress XAF1 as verified via Western Blot (Figure 6A). Control and XAF1-overexpressing SK-N-SH cells were subcutaneously engrafted into the left and right flanks of the mice respectively and tumor growth was monitored over a period of 33 days (Figure 6B and 6C). We observed that XAF1-overexpressing SK-N-SH cells (right flank) showed a significant reduction in tumor growth compared to control cells (left flank) (Figure 6B and 6C). In contrast to the effect of XAF1 knockdown, overexpression of XAF1 led to a decrease in tumor growth rate (Figure 6B and 6C). Consistent with our *in vitro* findings, these observations suggest that XAF1 plays an important role in tumor development whereby it may function as a tumor suppressor in neuroblastoma.

## DISCUSSION

In recent years, XAF1 being a potent antagonist of XIAP, has been studied with interest for its involvement in tumor suppression. XAF1 is poorly expressed in many cancers despite the lack of association with any inactivating mutation or deletion, suggesting that epigenetic silencing or upstream regulation of XAF1 could be the key to controlling its expression [13, 14]. In this study, we examined the expression of XAF1 in human neuroblastoma and found that XAF1 is either absent or lowly expressed in neuroblastomas. In current clinical practice, no molecular marker exists that has prognostic function in post-treatment samples. Only pre-treatment samples are tested and stratified accordingly. This poses a problem when diagnoses are established in places with incomplete molecular analyses or insufficient volume of specimen at the time of diagnosis, and only post-treatment samples are available. Our results imply that there may be prognostic value in analyzing the post-treatment samples for XAF1 as this marker demonstrates strong correlation between expression status and survival outcome in particular to post-treatment samples (Figure 2C and 2F). This suggests that XAF1 may be a potential tumor suppressor and prognostic marker whereby its low expression down-regulates an important apoptotic mechanism for tumor suppression.

Moreover, XAF1 was previously shown to be involved in mouse neuronal developmental apoptosis and cancers with neural crest origin such as melanoma and neuroblastoma [15]. Here, we found that overexpression of XAF1 is sufficient to induce significant apoptosis in neuroblastoma. Using sympathetic neuroblasts of the SCG, we also studied XAF1 relevance to neuronal death in the peripheral nervous system during mammalian development. During the first two weeks after birth, NGF is synthesized for the survival of neuronal progenitors, however, one-third of these cells which are out-competed for NGF, undergo apoptosis mediated by KIF1Bβ [8, 9, 19]. Here, we show that XAF1 is present in the sympathetic neuroblasts of SCG from newborn mouse pups and its expression is correspondingly reduced when KIF1Bβ is deleted, indicating KIF1Bβ as an upstream regulator of XAF1. Moreover, both KIF1Bβ and XAF1 are poorly expressed in adult SCG. In addition, using rat pheochromocytoma PC12 cells and primary sympathetic neuroblasts isolated from SCGs of P2 rat pups as a model for NGF withdrawal-dependent culling, we showed that XAF1 is indeed required for KIF1Bβ-mediated apoptosis. Upon NGF withdrawal, KIF1Bβ and XAF1 are induced with XAF1 demonstrating a more sustained induction, resulting in apoptosis. However, silencing of XAF1 rescued differentiated PC12 cells and isolated neuroblasts from NGF withdrawal-dependent apoptosis in spite of KIF1Bβ induction, providing support that XAF1 is a critical effector for apoptosis downstream of KIF1Bβ. This was confirmed in neuroblastoma cells whereby XAF1 knockdown in the presence of KIF1Bβ resulted in resistance to KIF1Bβ-mediated apoptosis.

Finally, we explored the importance of XAF1 expression in regulating tumor growth. Using mice xenografts, we showed that XAF1 knockdown increases tumor growth whereas XAF1 overexpression decreases tumor progression. XAF1 expression level is tightly linked to tumor development and increasing XAF1 may be imperative in delaying the growth of cancer cells. Together with our *in vitro* findings, our study suggests that XAF1 may function as a tumor suppressor in neuroblastoma.

In summary, we provided evidence to demonstrate the tumor suppressive effect and prognostic potential of XAF1 in neuroblastoma. Failure of apoptosis to appropriately cull neuronal progenitors during development predisposes to sympathetic nervous system tumors such as neuroblastoma. Improper alterations in downstream effectors of developmental apoptosis such as down-regulation of XAF1 may play a pathogenic role in neuroblastoma development by allowing neuronal progenitors to escape from developmental culling and thereby predisposing them to neoplastic transformation. Our findings provide support that XAF1 and its functions such as XIAP inhibition may be a promising pathway to target for neuroblastoma treatment and management. However, the exact mechanism underlying XAF1 tumor suppression and the prospective therapeutic strategies involving XAF1 remain to be characterized in neuroblastoma.

## MATERIALS AND METHODS

### Cell culture

Human neuroblastoma cell lines (SK-N-AS, SK-N-SH, NLF, CHP212, NB1) were maintained in RPMI containing 10% fetal bovine serum (FBS) (Hyclone) and 1% penicillin-streptomycin. PC12 cells were maintained in DMEM containing 5% FBS (Hyclone), 10% horse serum (Hyclone) and 1% penicillin-streptomycin. All cells were obtained from American Type Culture Collection (ATCC) and cultured in the presence of 5% CO_2_ at 37°C.

### Plasmids and shRNAs

Flag-XAF1 plasmid was purchased from GeneCopoeia. Lentivirus expressing XAF1 was produced from pLenti-XAF1 plasmid (GeneCopoeia). Flag-KIF1Bβ(600-1400) plasmid was generated as described previously [9]. shRNA-expressing lentiviral plasmids targeting XAF1 for lentivirus production were obtained from Mission shRNA (sigma). shRNA sequence targeting human XAF1 is (#94) sense oligos: 5′-CTAGACCGGGCTAGCCATATGCAGAGGATTCTCGAGAATCCTCTGCATATGGCTAGCTTTTTTGT-3′, (#94) antisense oligos: 5′-CTAGACAAAAAAGCTAGCCATATGCAGAGGATTCTCGAGAATCCTCTGCATATGGCTAGCCCGGT-3′, (#28) sense oligos: 5′- CTAGACCGGGTTCATCAAAGAAAGCACCAACTCGAGTTGGTGCTTTCTTTGATGAACTTTTTTGT-3′, (#28) antisense oligos: 5′-CTAGACAAAAAAGTTCATCAAAGAAAGCACCAACTCGAGTTGGTGCTTTCTTTGATGAACCCGGT-3′.

### Apoptotic assays

Apoptosis was assessed either via quantification of apoptotic nuclei using GFP-histone as previously described [8]. Alternatively, apoptotic cells were visualized and quantified by immunofluorescence staining for cleaved caspase-3. Apoptosis induction characterized by mitochondrial membrane potential changes was determined by fluorescence-activated cell sorting (FACS) analysis using tetramethylrhodamine ethyl ester (TMRE) staining. Neuronal apoptosis was assessed via quantification of apoptotic nuclei in Tuj1-positive neuroblasts. Statistical analysis was performed by Student's t-test unless stated otherwise.

### NGF withdrawal assay

Undifferentiated PC12 cells were plated onto collagen IV-coated plates (Corning) and differentiated in culture medium containing 50 ng/ml NGF (Accurate Chemical) for 5 – 7 days. Primary sympathetic neuroblasts isolated from SCGs of post-gestation rat pups (P2) were cultured as previously described [20]. Differentiated PC12 cells and neuroblasts were infected with lentivirus encoding shXAF1 or nontargeting control virus (shSCR) followed by NGF withdrawal with anti-NGF antibody (1:5000, Sigma) for up to 48 hours where they were harvested for subsequent analysis.

### Immunoblot analysis

Cells were lysed in EBC buffer (50 mM Tris at pH 8.0, 120 mM NaCl, 0.5% NP-40) containing protease inhibitors. Equal amount of protein, as quantified via the Bradford assay, were separated by SDS-polyacrylamide gel electrophoresis and transferred onto PVDF membrane (Bio-Rad). Blots were incubated with specific primary antibodies as follows: Rabbit polyclonal anti-KIF1Bβ was a gift from Dr. Susanne Schlisio. Rabbit polyclonal anti-Flag and rabbit monoclonal cleaved caspase-3 (Asp175) (5A1E) were purchased from Cell Signaling Technology. Mouse monoclonal β-actin (C4) was purchased from Santa Cruz Biotechnology. Primary antibody signals were detected using either anti-rabbit or anti-mouse horseradish peroxidase-conjugated secondary antibodies (Cell Signaling Technology).

### Immunofluorescence and confocal microscopy

Cells were grown on cover slips, fixed in 4% paraformaldehyde (PFA), quenched with 10 mmol/L glycine, permeabilized with 0.1% Triton X-100, and blocked with 5% goat serum. Cells were then incubated with primary antibodies (1:1000) in phosphate-buffered saline (PBS) containing 0.1% bovine serum albumin (BSA) overnight at 4°C, followed by 1 hour incubation with secondary antibodies conjugated to fluorophores (Alexa Fluor® 488 purchased from Life Technologies) (1:1000) in PBS with 0.1% BSA. All steps were interspersed with two washes with PBS or PBS with 0.1% BSA and performed at room temperature unless otherwise stated. Cover slips were then mounted onto glass slides using Vectashield mounting medium containing DAPI (Vector Laboratories). All images were acquired using a confocal laser imaging system (FluoView FV1000, Olympus) and analyzed with Olympus Fluoview Ver. 1.7c software. Images were captured using UPLAPO 100X/1.35 NA Oil objective with a 2X zoom and the following settings – Frame Size: 1024; Scan Speed: 4; Acquisition: 12-bit; Line Averaging Mode: 2; and Pinhole: 180μm. The excitation wavelengths were 488nm and 405nm for Alexa Fluor® 488 and DAPI respectively.

### Tissue microarray (TMA) and immunohistochemistry

TMA analysis was performed on 86 neuroblastoma patients' samples obtained from KK Women's and Children's Hospital. Ethical permission was obtained from the SingHealth Central Institutional Review Board. TMA of tumor were constructed in triplicate from formalin-fixed paraffin-embedded tissue blocks using a 1 mm-wide diameter punch (Estigen) and a manual tissue-arraying instrument (Beecher Instruments). 4 μm-thick unstained sections of TMA blocks were treated with high pH H2 buffer (Leica Biosystem) for 20 minutes and stained with anti-XAF1 antibody (HPA057302) (Sigma) at dilution 1:250. DAB substrate was used as the chromogen and nuclei were counterstained with hematoxylin.

### Generation of conditional KIF1Bβ Knock-out

Conditional KIF1Bβ Knock-out (KO) mouse was generated by Dr. Susanne Schlisio's laboratory as described previously [21]. Briefly, the constitutive KO alleles specific for KIF1Bβ-isoform were obtained by crossing transgenic mice expressing Dopamine β-hydroxylase-Cre recombinase (DBH-Cre) with mice expressing floxed-targeting construct, which will result in the deletion of exon 22 and the subsequent loss of KIF1Bβ-isoform function [22]. Primary sympathetic neuroblasts from the SCG of post-gestation conditional KIF1Bβ KO mouse pups (P0 to P2) were isolated for further immunoblot analysis. Mouse primary sympathetic neuroblasts from P0 to P2 floxed-mice were isolated from SCG and cultured as described previously [20]. The neuroblasts were transduced with Ad-Cre adenovirus to abolish KIF1Bβ expression *ex vivo*, and followed by immunoblot analysis.

### Mice xenografts for XAF1 knockdown and overexpression

10 athymic female mice (CrTac:NCr-*Foxn1^nu^*), 4 – 6 weeks old, (18.5 – 24.7 g) were purchased from InVivos Pte Ltd. All experiments are performed under the approval of the Institutional Animal Care and Use Committee (IACUC) in compliance with the law and guidelines stated. CHP212 neuroblastoma cells were stably transfected with plasmids encoding sh*XAF1* (94) followed by infection with luciferase-expressing lentivirus. 1 × 10^6^ of cells stably expressing XAF1 knockdown or control cells were injected subcutaneously into the right and left flanks of 5 mice respectively. Tumor analysis was performed by intraperitoneal injection of D-luciferin solution (150 mg/kg) (PerkinElmer) into the mice and imaged with an IVIS spectrum. Bioluminescent signals were then quantified using Living Imaging 4.4 (Caliper Life Sciences). SK-N-SH neuroblastoma cells were transduced with luciferase lentivirus. The cells were selected, expanded and transduced with either control virus or lentivirus-encoding XAF1. Subsequently, 1 × 10^6^ of transduced cells (control or XAF1-overexpressing) was injected subcutaneously into the left and right flanks of 5 mice respectively. Mice were imaged and tumor analysis was performed as described above.

### SUPPLEMENTARY METHODS




