# Supplementary material for: XAF1 promotes neuroblastoma tumor suppression and is required for KIF1Bβ-mediated apoptosis
Source: Oncotarget. 2016 Apr 15;7(23):34229–39. doi: 10.18632/oncotarget.8748 (PMC5085151; doi:10.18632/oncotarget.8748)
Supplement: Supplementary file 1 [file oncotarget-07-34229-s001.pdf]

XAF1 promotes neuroblastoma tumor suppression and is required for KIF1Bβ-mediated apoptosis

SUPPLEMENTARY METHODS

Survival curves

A senior consultant pediatric pathologist who was blinded to the clinical information scored the staining intensity (0 – 3+). Survival outcomes were obtained from the Singapore Childhood Cancer Registry and analyzed using the Kaplan-Meier method. The log-rank test was used to compare survival differences between subgroups with absent to low (0 and 1+) and moderate to high staining (2+ and 3+).

SUPPLEMENTARY FIGURES AND TABLES

A

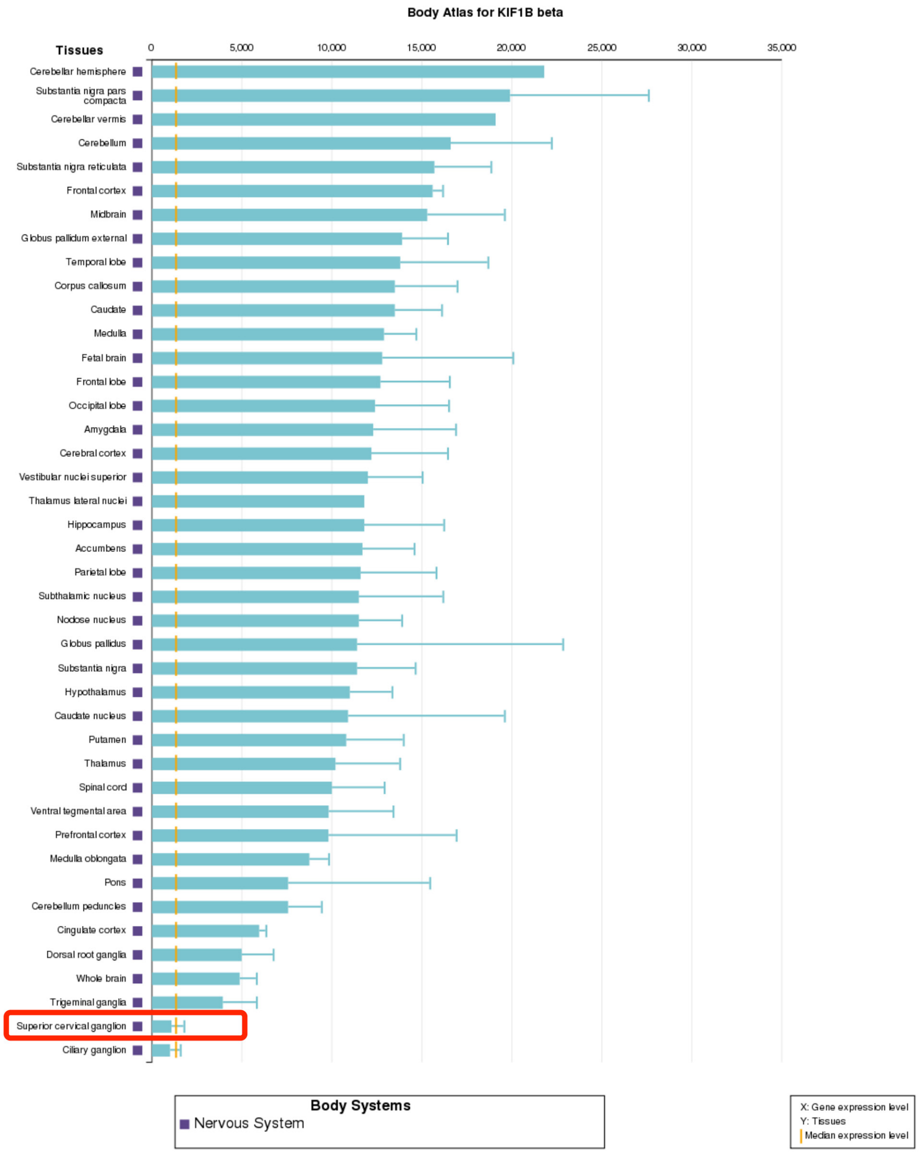

Supplementary Figure S1, related to Figure 1: XAF1 and KIF1Bβ expression is low in superior cervical ganglia (SCG) compared to other tissues of the adult nervous system. A. XAF1 gene expression. (*Continued*)

B

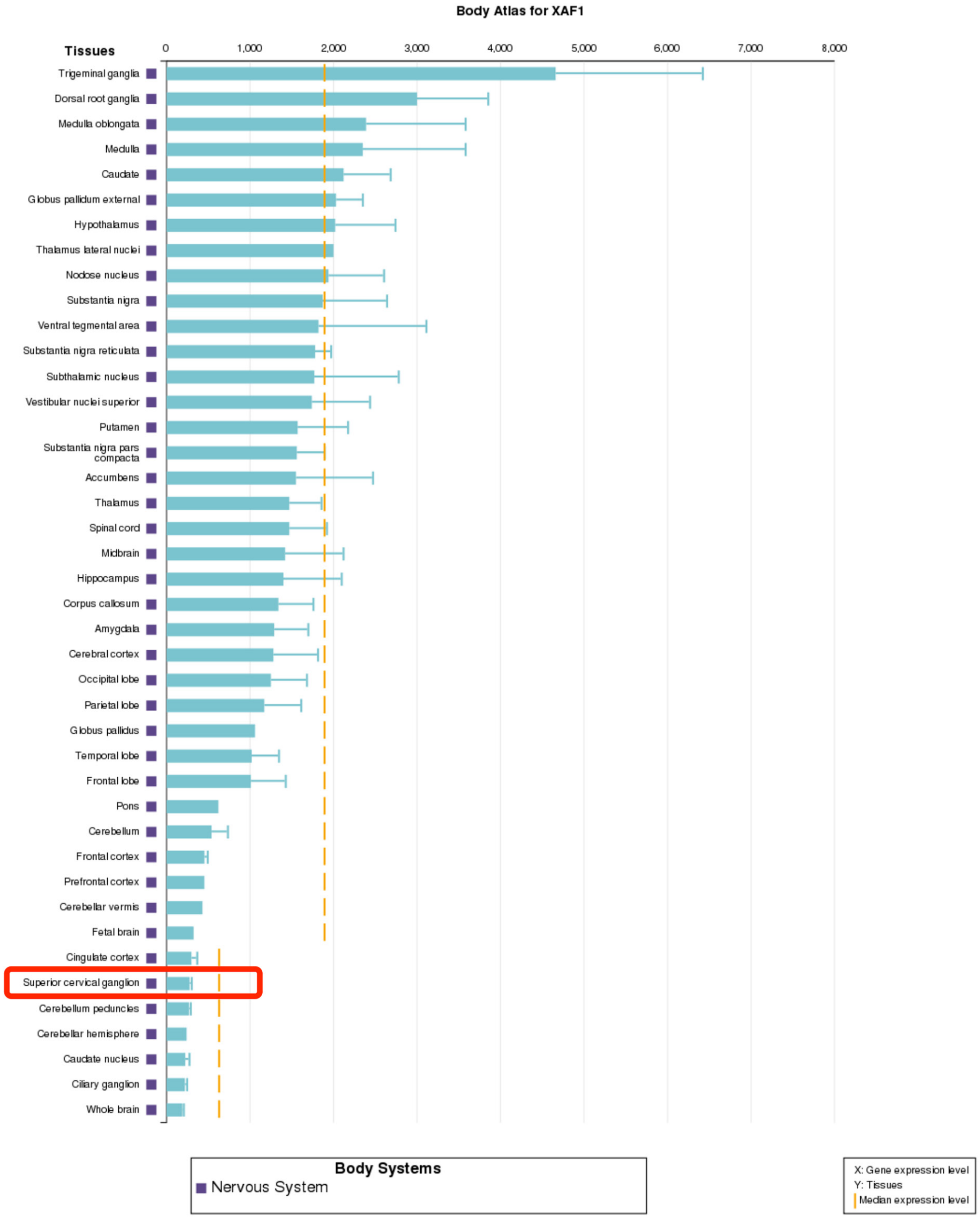

Supplementary Figure S1 related to Figure 1: (*Continued*), XAF1 and KIF1Bβ expression is low in superior cervical ganglia (SCG) compared to other tissues of the adult nervous system. B. KIF1Bβ gene expression in SCG relative to other tissues of the nervous system using microarray-based RNA expression datasets from NextBio database (http://www.nextbio.com/b/nextbio.nb).

A

XAF1 Expression	Alive cases with known disease status	Alive with Disease
Null (0)	13	6
Low (1+)	16	
Moderate (2+)	8	1
High (3+)	1	
Total	38	7

B

XAF1 Expression	Alive post-treatment cases with known disease status	Alive with Disease
Null (0)	6	4
Low (1+)	10	
Moderate (2+)	5	0
High (3+)	1	
Total	22	4

C

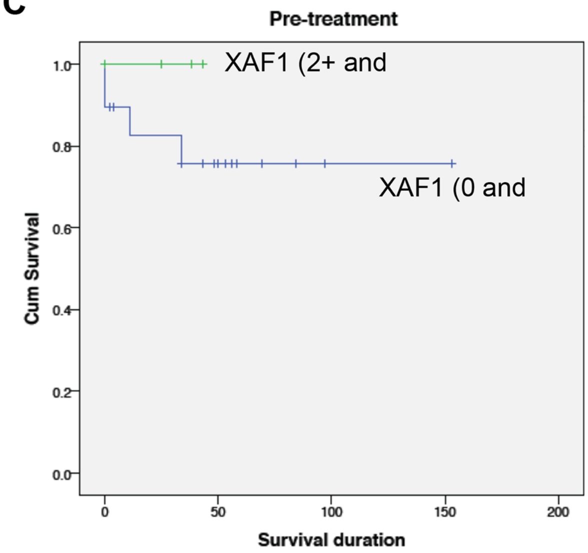

Supplementary Figure S2, related to Figure 2: XAF1 expression is correlated to survival outcomes in human neuroblastomas. A. Association between XAF1 expression and patients alive with known disease status. **B.** Association between XAF1 expression and post-treatment patients alive with known disease status. **C.** Kaplan-Meier survival curves for patients with complete absence and low expression of XAF1 (0 and 1+) compared to moderate and high expression of XAF1 (2+ and 3+) in pre-treatment cases.

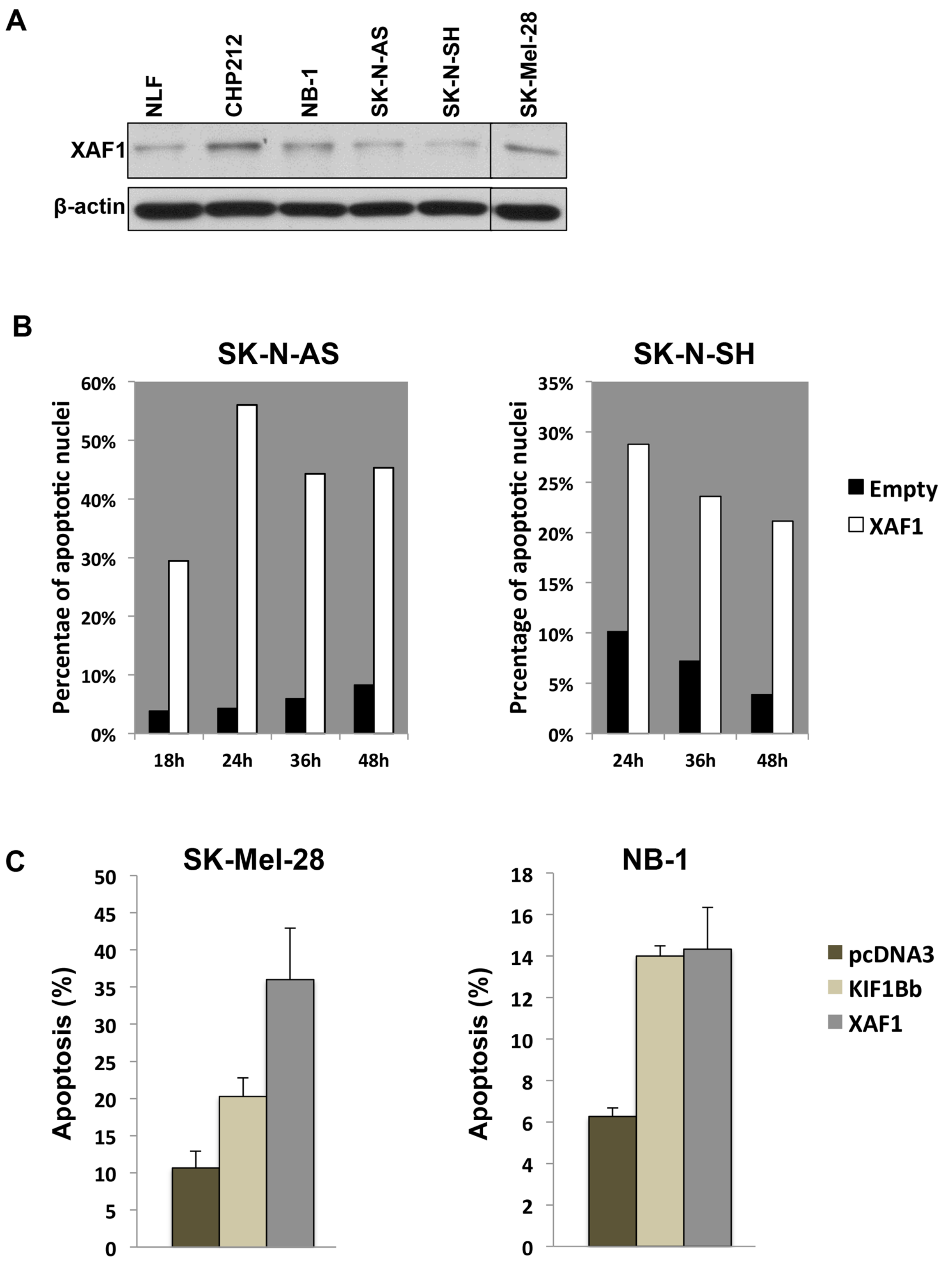

Supplementary Figure S3, related to Figure 3 and 4: Overexpression of XAF1 increases apoptosis *in vitro*. A. Basal expression of XAF1 in a panel of neuroblastoma and melanoma cell lines. **B.** Percentage of apoptotic cells quantified by GFP-positive nuclei exhibiting apoptotic changes after co-transfection with plasmids encoding GFP-histone and Flag-XAF1 or empty vector plasmids for 18hr, 24hr, 36hr and 48hr. **C.** Percentage of apoptosis in NB-1 neuroblastoma and SK-Mel-28 melanoma cells after transfection with KIF1Bβ or XAF1 as determined by fluorescence-activated cell sorting (FACS) analysis using TMRE staining.

Supplementary Table S1, related to Figure 2: Neuroblastoma patients' data on specimen source, XAF1 expression, 1p-, 17q- and MYCN status, clinical stage, treatment status and outcome

See Supplementary File 1
