# Supplementary material for: XAF1 promotes neuroblastoma tumor suppression and is required for KIF1Bβ-mediated apoptosis
Source: Oncotarget. 2016 Apr 15;7(23):34229–39. doi: 10.18632/oncotarget.8748 (PMC5085151; doi:10.18632/oncotarget.8748)
Supplement: Supplementary file 2 [file oncotarget-07-34229-s002.docx]

**Supplementary table:**

**Table S1, related to Figure 2.**

**Neuroblastoma patients’ data on specimen source, XAF1 expression, 1p-, 17q- and MYCN status, clinical stage, treatment status and outcome.**

| **Patient no** | **Specimen source** | **Treatment status** | **XAF1 IHC** | **1p status** | **17q status** | **MYCN** | **INSS Stage** | **COG Risk** | **Present status** | **Disease status** | **Survival duration (years)** |
| --- | --- | --- | --- | --- | --- | --- | --- | --- | --- | --- | --- |
| 1 | Primary | Pre-treatment | 0 | Deleted | Gain | Not amplified | 3 | Intermediate | Alive | Alive with Disease | 12.81 |
| 2 | Primary | Post-treatment | 1+ | Intact | Gain | Not amplified | 4 | High | Alive | No Evidence of Disease | 11.95 |
| 3 | Primary | Post-treatment | 0 | Intact | Unknown | Not amplified | 4 | High | Alive | Unknown | 0.05 |
| 4 | Primary | Post-treatment | 1+ | Intact | Gain | Not amplified | 4 | High | Alive | Unknown | 0.06 |
| 5 | Primary | Post-treatment | 0 | Deleted | No gain | Not amplified | 4 | Intermediate | Alive | No Evidence of Disease | 7.64 |
| 6 | Primary | Post-treatment | 0 | Deleted | Unknown | Amplified | 4 | High | Alive | Unknown | 0.06 |
| 7 | Primary | Pre-treatment | 0 | Deleted | Gain | Amplified | 4 | High | Alive | No Evidence of Disease | 8.13 |
| 8 | Primary | Post-treatment | 0 | Intact | Gain | Not amplified | 4 | High | Dead | Death from Disease | 1.15 |
| 9 | Primary | Post-treatment | 0 | Intact | Gain | Not amplified | 4 | High | Dead | Death from Disease | 1.83 |
| 10 | Primary | Post-treatment | 0 | Deleted | No gain | Not amplified | 4 | High | Dead | Death from Disease | 2.27 |
| 11 | Primary | Pre-treatment | 0 | Intact | Unknown | Not amplified | 1 | Low | Alive | Unknown | 0.01 |
| 12 | Primary | Pre-treatment | 0 | Deleted | No gain | Not amplified | 4 | High | Alive | Unknown | 0.02 |
| 13 | Primary | Post-treatment | 0 | Intact | Gain | Not amplified | 4 | High | Dead | Death from Disease | 0.78 |
| 14 | Primary | Post-treatment | 0 | Intact | Unknown | Not amplified | 4 | High | Alive | No Evidence of Disease | 0.78 |
| 15 | Metastasis | Post-treatment | 0 | Intact | Unknown | Not amplified | 4 | High | Dead | Death from Disease | 1.29 |
| 16 | Primary | Post-treatment | 1+ | Intact | Gain | Amplified | 4 | High | Dead | Death from Disease | 0.68 |
| 17 | Primary | Post-treatment | 1+ | Deleted | Unknown | Amplified | 4 | High | Dead | Death from Disease | 1.22 |
| 18 | Primary | Post-treatment | 1+ | Intact | No gain | Not amplified | 4 | High | Alive | No Evidence of Disease | 4.98 |
| 19 | Primary | Post-treatment | 1+ | Intact | Unknown | Amplified | 3 | High | Alive | LTFU / Unknown | 1.67 |
| 20 | Primary | Pre-treatment | 1+ | Intact | No gain | Not amplified | 2 | Low | Alive | Alive with Disease | 4.72 |
| 21 | Primary | Post-treatment | 1+ | Intact | Gain | Not amplified | Unknown | Unknown | Dead | Death from Disease | 0.90 |
| 22 | Primary | Pre-treatment | 0 | Intact | No gain | Not amplified | 2 | Low | Alive | No Evidence of Disease | 4.25 |
| 23 | Primary | Post-treatment | 1+ | Deleted | Unknown | Amplified | 4 | High | Alive | LTFU / Unknown | 0.26 |
| 24 | Primary | Post-treatment | 1+ | Intact | No gain | Not amplified | 3 | High | Alive | No Evidence of Disease | 3.96 |
| 25 | Metastasis | Post-treatment | 0 | Intact | Unknown | Not amplified | 4 | High | Dead | Death from other causes | 1.18 |
| 26 | Primary | Post-treatment | 1+ | Intact | Gain | Not amplified | Unknown | Unknown | Alive | LTFU / Unknown | 0.02 |
| 27 | Primary | Post-treatment | 1+ | Intact | Unknown | Not amplified | 4 | High | Dead | Death from Disease | 1.89 |
| 28 | Primary | Post-treatment | 1+ | Intact | Gain | Not amplified | 3 | Unknown | Alive | LTFU / Unknown | 1.98 |
| 29 | Primary | Pre-treatment | 0 | Intact | No gain | Not amplified | 3 | High | Dead | Death from Disease | 2.86 |
| 30 | Metastasis | Post-treatment | 2+ | Deleted | Gain | Amplified | 4 | High | Dead | Death from Disease | 1.87 |
| 31 | Metastasis | Post-treatment | 1+ | Intact | Gain | Not amplified | 2 | Low | Alive | No Evidence of Disease | 3.62 |
| 32 | Primary | Pre-treatment | 1+ | Intact | Gain | Not amplified | Unknown | Intermediate | Alive | No Evidence of Disease | 3.60 |
| 33 | Primary | Post-treatment | 1+ | Intact | Unknown | Not amplified | 3 | Unknown | Alive | LTFU / Unknown | 0.36 |
| 34 | Primary | Post-treatment | 1+ | Intact | Unknown | Not amplified | 4 | Intermediate | Alive | Alive with Disease | 1.99 |
| 35 | Primary | Post-treatment | 1+ | Deleted | Unknown | Not amplified | 4 | High | Alive | LTFU / Unknown | 0.01 |
| 36 | Primary | Post-treatment | 2+ | Intact | Unknown | Not amplified | 4 | High | Dead | Death from Disease | 0.15 |
| 37 | Primary | Post-treatment | 2+ | Intact | Unknown | Not amplified | 4 | High | Alive | LTFU / Unknown | 1.97 |
| 38 | Primary | Post-treatment | 0 | Intact | Unknown | Not amplified | 4 | High | Alive | No Evidence of Disease | 3.80 |
| 39 | Primary | Post-treatment | 2+ | Deleted | Gain | Amplified | 4 | High | Alive | No Evidence of Disease | 3.19 |
| 40 | Primary | Post-treatment | 1+ | Deleted | Unknown | Not amplified | 4 | High | Alive | LTFU / Unknown | 0.02 |
| 41 | Primary | Post-treatment | 1+ | Intact | Gain | Not amplified | 3 | Intermediate | Alive | Alive with Disease | 1.42 |
| 42 | Primary | Post-treatment | 2+ | Intact | Unknown | Not amplified | 4 | Intermediate | Alive | No Evidence of Disease | 3.00 |
| 43 | Metastasis | Post-treatment | 1+ | Intact | Unknown | Not amplified | 3 | Unknown | Alive | No Evidence of Disease | 2.22 |
| 44 | Metastasis | Post-treatment | 1+ | Intact | Unknown | Not amplified | Unknown | Unknown | Alive | LTFU / Unknown | 0.07 |
| 45 | Metastasis | Post-treatment | 1+ | Deleted | Unknown | Amplified | 5 | High | Dead | Death from Disease | 0.82 |
| 46 | Metastasis | Post-treatment | 1+ | Intact | Unknown | Amplified | 4 | High | Alive | Alive with Disease | 0.20 |
| 47 | Primary | Post-treatment | 1+ | Deleted | Unknown | Amplified | Unknown | High | Dead | Death from Disease | 0.10 |
| 48 | Primary | Post-treatment | 1+ | Not done | Unknown | Amplified | 4 | High | Alive | No Evidence of Disease | 1.44 |
| 49 | Primary | Post-treatment | 1+ | Intact | Unknown | Not amplified | 3 | High | Alive | LTFU / Unknown | 0.82 |
| 50 | Metastasis | Post-treatment | 1+ | Not done | Unknown | Amplified | Unknown | High | Dead | Death from Disease | 0.82 |
| 51 | Metastasis | Pre-treatment | 1+ | Not done | Unknown | Not amplified | 4 | High | Dead | Death from Disease | 0.00 |
| 52 | Primary | Pre-treatment | 0 | Not done | Unknown | Unknown | 2 | Unknown | Alive | No Evidence of Disease | 7.08 |
| 53 | Primary | Post-treatment | 2+ | Not done | No gain | Not amplified | 3 | Intermediate | Alive | No Evidence of Disease | 7.02 |
| 54 | Primary | Unknown | 0 | Not done | Unknown | Amplified | 4 | High | Alive | No Evidence of Disease | 6.70 |
| 55 | Primary | Post-treatment | 0 | Not done | Unknown | Amplified | Unknown | High | Alive | Alive with Disease | 5.28 |
| 56 | Primary | Post-treatment | 1+ | Not done | Unknown | Not amplified | 4 | Unknown | Alive | No Evidence of Disease | 6.10 |
| 57 | Primary | Post-treatment | 1+ | Not done | Unknown | Amplified | 4 | High | Dead | Death from Disease | 0.76 |
| 58 | Primary | Pre-treatment | 1+ | Not done | Unknown | Not amplified | Unknown | Unknown | Alive | No Evidence of Disease | 5.80 |
| 59 | Primary | Post-treatment | 1+ | Not done | Unknown | Unknown | Unknown | Unknown | Dead | Death from other causes | 0.28 |
| 60 | Primary | Post-treatment | 0 | Not done | Unknown | Amplified | 4 | High | Alive | No Evidence of Disease | 5.47 |
| 61 | Metastasis | Post-treatment | 0 | Not done | Unknown | Not amplified | 4 | High | Alive | LTFU / Unknown | 0.06 |
| 62 | Primary | Pre-treatment | 0 | Not done | Unknown | Amplified | Unknown | Unknown | Alive | LTFU / Unknown | 0.42 |
| 63 | Primary | Pre-treatment | 1+ | Not done | Unknown | Not amplified | 4 | High | Alive | LTFU / Unknown | 0.21 |
| 64 | Primary | Post-treatment | 1+ | Not done | Unknown | Amplified | 2 | High | Alive | LTFU / Unknown | 2.32 |
| 65 | Primary | Post-treatment | 2+ | Not done | Unknown | Not amplified | 1 | Low | Alive | No Evidence of Disease | 5.80 |
| 66 | Primary | Pre-treatment | 1+ | Not done | Unknown | Unknown | 1 | Low | Alive | No Evidence of Disease | 4.47 |
| 67 | Primary | Post-treatment | 2+ | Not done | Unknown | Unknown | 4 | High | Alive | LTFU / Unknown | 1.67 |
| 68 | Primary | Pre-treatment | 1+ | Not done | Unknown | Unknown | 3 | Unknown | Dead | Death from Disease | 0.92 |
| 69 | Primary | Pre-treatment | 0 | Not done | Unknown | Not amplified | Unknown | Unknown | Alive | No Evidence of Disease | 4.90 |
| 70 | Primary | Post-treatment | 0 | Not done | Unknown | Not amplified | 4 | High | Alive | LTFU / Unknown | 0.44 |
| 71 | Primary | Post-treatment | 2+ | Not done | Unknown | Not amplified | Unknown | Unknown | Alive | LTFU / Unknown | 1.48 |
| 72 | Primary | Pre-treatment | 2+ | Not done | Unknown | Unknown | 1 | Low | Alive | No Evidence of Disease | 3.64 |
| 73 | Primary | Post-treatment | 0 | Not done | Unknown | Not amplified | 3 | Intermediate | Alive | No Evidence of Disease | 4.30 |
| 74 | Primary | Post-treatment | 1+ | Not done | Unknown | Not amplified | 3 | Unknown | Alive | LTFU / Unknown | 0.57 |
| 75 | Primary | Pre-treatment | 0 | Not done | Unknown | Not amplified | 2 | Low | Alive | No Evidence of Disease | 4.03 |
| 76 | Primary | Post-treatment | 1+ | Not done | Gain | Not amplified | 3 | Intermediate | Dead | Death from other causes | 1.04 |
| 77 | Primary | Pre-treatment | 1+ | Not done | Unknown | Not amplified | Unknown | Unknown | Dead | Death from Disease | 0.00 |
| 78 | Metastasis | Post-treatment | 1+ | Not done | Unknown | Not amplified | 4 | High | Dead | Death from Disease | 0.29 |
| 79 | Primary | Post-treatment | 1+ | Not done | Unknown | Not amplified | 4 | High | Alive | LTFU / Unknown | 0.22 |
| 80 | Metastasis | Post-treatment | 1+ | Not done | Unknown | Not amplified | 4 | High | Alive | LTFU / Unknown | 0.33 |
| 81 | Primary | Post-treatment | 3+ | Not done | Unknown | Not amplified | 3 | Intermediate | Alive | No Evidence of Disease | 4.50 |
| 82 | Primary | Pre-treatment | 1+ | Not done | No gain | Not amplified | 1 | Low | Alive | No Evidence of Disease | 2.91 |
| 83 | Primary | Pre-treatment | 2+ | Not done | Gain | Not amplified | 1 | Low | Alive | No Evidence of Disease | 3.23 |
| 84 | Primary | Pre-treatment | 2+ | Not done | Unknown | Not amplified | Unknown | Unknown | Alive | LTFU / Unknown | 0.02 |
| 85 | Primary | Pre-treatment | 2+ | Not done | No gain | Not amplified | 1 | Low | Alive | Alive with Disease | 2.15 |
| 86 | Primary | Post-treatment | 2+ | Not done | Unknown | Amplified | 4 | High | Alive | No Evidence of Disease | 2.42 |
